# Association between single-nucleotide polymorphisms within candidate genes and fertility in Landrace and Duroc pigs

**DOI:** 10.1186/s13028-019-0493-x

**Published:** 2019-12-03

**Authors:** Nina Hårdnes Tremoen, Maren Van Son, Ina Andersen-Ranberg, Eli Grindflek, Frøydis Deinboll Myromslien, Ann Helen Gaustad, Dag Inge Våge

**Affiliations:** 1 Department of Biotechnology, Inland Norway University of Applied Sciences, 2318 Hamar, Norway; 2 Norsvin, Storhamargata 44, 2317 Hamar, Norway; 3 Department of Animal and Aquacultural Sciences, Centre for Integrative Genetics (CIGENE), Faculty of Biosciences, Norwegian University of Life Sciences, 1432 Ås, Norway

**Keywords:** Boar, Candidate genes, Fertility, SNP

## Abstract

Finding effective predictors of traits related to boar fertility is essential for increasing the efficiency of artificial insemination systems in pig breeding. The objective of this study was to find associations between single-nucleotide polymorphisms (SNPs) within candidate genes and fertility in the breeds Landrace and Duroc. Animals with breeding values for total number of piglets born, were re-sequenced for exonic regions of 14 candidate genes related to male and female fertility using samples from 16 Landrace boars and 16 Duroc boars (four with high and four with low breeding value of total number of piglets born for each breed for male fertility, and the same for female fertility) to detect genetic variants. Genotyping for the detected SNPs was done in 619 Landrace boars and 513 Duroc boars. Two SNPs in *BMPR1* and one SNP in *COX-2* were found significantly associated with the total number of piglets born in Landrace. In Duroc, two SNPs in *PLC*_*z*_, one SNP in *VWF* and one SNP in *ZP3* were found significantly associated with total number of piglets born. These SNPs explained between 0.27% and 1.18% of the genetic variance. These effects are too low for being used directly for selection purposes but can be of interest in SNP-panels used for genomic selection.

## Findings

The traditional measurements of boar fertility are pregnancy rate and litter size. However, these measurements are retrospective and highly influenced by the breeding management and the genetic potential of the sows. Finding effective predictors of traits related to boar fertility would allow for excluding the less fertile boars from pig production and improving the breeding system.

Several genes have been proposed as candidate genes for male fertility, including phospholipase C zeta (*PLCz)* and cyclooxygenase isoenzyme type 2 (*COX-2*) [1]. Also, when it comes to female fertility, several genes are involved such as bone morphogenic protein 15 (*BMP15)*, bone morphogenic protein receptor 1B (*BMPR1B),* and growth differentiation factor 9 (*GDF9*) [2-4]. The estrogen receptors 1 and 2 (*ESR1* and *ESR2*) have proved to be involved in both male and female reproduction [5, 6]. Samples from sows were not included in the current study, but genes linked to female reproduction were included to investigate the boars as maternal grandsires. Moreover, all the genes were analysed in both breeds.

In this study, we analysed male fertility of the boars as sire of their litters, and female fertility measured as fertility of the boars’ daughters for detection of genetic variants. The variance components were estimated and the heritabilities were estimated by dividing female or male variance by the total variance. The estimated variance components and estimated breeding values (EBVs) for total number of piglets born (TNB) were calculated using a univariate animal repeatability model and included both the direct genetic effect (male fertility) and sire effect based on daughter fertility (female fertility). In addition, herd × year, parity to the mother (parity_m), parity of the sow (parity), season as fixed effects, age of sow at farrowing within parity as fixed regression effects (age(parity), age^2^(parity)) and litter and animal (ID, ID_sire) as random effects were included in the following model:

$${\text{y}} = {\text{herd}} \times {\text{year}} + {\text{parity}}\_{\text{m}} + {\text{parity}} + {\text{season}} + {\text{age}}({\text{parity}}) + {\text{age}}^{2} ({\text{parity}}) + {\text{litter}} + {\text{ID}} + {\text{ID}}\_{\text{sire}} + \rm{e}$$

Only results from purebred litters were used to estimate (co-)variance components and breeding values, and 86,539 litters from Landrace and 16,819 litters from Duroc had records on TNB. Pedigrees were traced back seven generations and included 61,293 and 13,480 animals for Landrace and Duroc, respectively.

The candidate genes were chosen based on previous reported involvement in fertility or in pathways related to reproduction. In total, 14 candidate genes were selected (Additional file 1), seven specific for male fertility, five specific for female fertility and two important for both male and female fertility. The coding regions of 14 genes were sequenced using samples from 16 Landrace and 16 Duroc boars selected based on their EBVs, to identify genetic variation in these candidate genes. Eight Landrace boars and eight Duroc boars (four with high and four with low breeding value for each breed) were selected based on male fertility. The other group (with equal number of animals) was selected based on female fertility. DNA was isolated from sperm cells at BioBank, Hamar, Norway using the Maxwell 16 Tissue DNA Purification Kit (Promega, Madison, WI, USA) and polymerase chain reaction (PCR) primers were designed to amplify the exons of the selected genes using Primer3 [7] (for primer sequences, see Additional file 2). PCR was performed using HOT FIREPol DNA Polymerase (Solis BioDyne, Tartu, Estonia) and analysed on ABI 3130XL (Applied Biosystems, Foster City, CA, USA). The PCR products were sequenced using the BigDye® Terminator v3.1 kit (Applied Biosystems) analyzed on ABI 3130XL (Applied Biosystems). Resulting sequences were aligned and screened for SNPs using the programs phred, phrap and consed [8, 9]. Out of the 14 re-sequenced genes, primers for 52 SNPs were designed. Additionally, one extra SNP (Von Willenbrand factor, *VWF*) was added to the analysis based on previous results [10]. SNPs were filtered based on call rate (> 0.97), minor allele frequency (MAF) (> 0.01) and deviation from Hardy–Weinberg equilibrium (HWE < 0.0001), leaving 36 SNPs for genotyping.

For the association study of male and female fertility, 619 Landrace boars and 513 Duroc boars were included. All the boars in this study have been used in AI during the last 10 years, with 107,640 litters in Landrace (mean TNB = 13.7 ± 3.6) and 16,849 litters in Duroc (mean TNB = 9.3 ± 2.9). The EBVs of direct genetic effect (male fertility) and maternal genetic effect (female fertility) are the phenotypes used for association. Genomic DNA was extracted from blood or semen using BioSprint DNA Kit (Qiagen, Hilden, Germany), assay design was done using Assay Design Suite software (Agena Biosciences, San Diego, CA, USA), and genotyping was done using the Sequenom massARRAY platform (Agene Biosciences) at CIGENE, Norwegian University of Life Sciences. Newly detected SNPs were submitted to the European Variant Archive (EVA) (https://www.ebi.ac.uk/eva) with accession number PRJEB23828.

Genotype effects were estimated using the following animal model in ASReml (v. 3.0.22.2):

$${\text{y}} =\upmu + {\text{SNP}} + {\text{ID}} + {\text{e}}$$

where y is EBV for TNB (male or female fertility), SNP genotype (AA, AB or BB) was fitted as a fixed effect and animal ID was fitted as a random effect. A pedigree-based relationship matrix was used, and a P-value < 0.05 was considered significant. The genetic variance explained by a SNP was calculated in ASReml as 2p(1− p)α^2^, where α is the allele substitution effect, divided by the additive genetic variance.

In Landrace, the analyses showed two significant SNPs, *rs331082315* in *BMPR1* and *rs337596396* in *COX-2* (male fertility) (Table 1), and two significant SNPs, *rs45435443* in *BMPR1* and *rs337596396* in *COX-2* (female fertility) (Table 2). In Duroc, the analyses resulted in three significant SNPs, *rs338483233* in *PLC*_*z*_, *rs328291649* in *VWF* and *rs339149260* in *ZP3* (male fertility) (Table 1) and two significant SNPs, *rs338483233* and *rs196952431* in *PLC*_*z*_ (female fertility) (Table 2). All the significant SNPs had previously been annotated in the pig genome.

**Table 1:** Significant SNPs for total number of piglets born as a male fertility trait

Breed	NL	NL	ND	ND	ND
Gene	*BMPR1B*	*COX-2*	*ZP3*	*PLC*_*z*_	*VWF*
Genbank	NW_003610967.1	NW_003300644.3	NM_213893.1	NM_214350.1	NM_001246221.1
Chromosome	8	9	3	5	5
Position	133743941	140252549	9840522	57682420	67005497
Genotype	[C/T]	[C/T]	[G/T]	[A/G]	[C/T]
SNP database	*rs331082315*	*rs337596396*	*rs339149260*	*rs338483233*	*rs328291649*
Genetic variance (%)	0.92	43,101	0. 55	0.71	0. 91
P-value	0.010	0.010	0.018	0.025	0.010
Minor allele frequency (MAF)	0.21	0.48	0.05	0.44	0.43
Hardy–Weinberg equilibrium (HWE)	0.58	0.78	0.19	0.89	0.74
Consequence	3 prime UTR variant	3 prime UTR variant	Intron variant	Missense variant	Missense variant

The significant SNPs for Landrace (NL) and Duroc (ND) boars based on their performance as father (male fertility). Positions are given according to pig genome build *Sscrofa10.2* obtained from the ENSEMBL web site (https://www.ensembl.org)

**Table 2:** Significant SNPs for total number of piglets born as a female fertility trait

Breed	NL	NL	ND	ND
Gene	*BMPR1B*	*COX-2*	*PLC*_*z*_	*PLC*_*z*_
Genbank	NW_003610967.1	NW_003300644,3	NM_214350.1	NM_214350.1
Chromosome	8	9	5	5
Position	133765484	140252549	57682420	57693191
Genotype	[A/G]	[C/T]	[A/G]	[C/T]
SNP database	*rs45435443*	*rs337596396*	*rs338483233*	*rs196952431*
Genetic variance (%)	0.27	0.67	0.82	0.71
P-value	0.039	0.042	0.016	0.030
Minor allele frequency (MAF)	0.05	0.48	0.44	0.43
Hardy–Weinberg equilibrium (HWE)	0.82	0.78	0.89	0.62
Consequence	Synonymous variant	3 prime UTR variant	Missense variant	Synonymous variant

The significant SNPs for Landrace (NL) and Duroc (ND) boars based on the performance of their daughters (female fertility). Positions are given according to pig genome build *Sscrofa10.2* obtained from the ENSEMBL web site (https://www.ensembl.org)

For Landrace, the SNP in *BMPR1B* was found significantly associated for both female and male fertility, meaning that this gene might be important for both male and female fertility traits. The gene family member *BMP1* has previously been found differentially expressed in both Landrace and Duroc boars with high/low levels of sperm DNA fragmentation [10] and several BMPs have been found important for fertility earlier [11].

Both *PLC*_*z*_ and *COX-2* are involved in spermatogenesis through the prostaglandin production and significant association between polymorphisms within the *COX-2* gene and litter size has previously been reported [1, 12]. In this study, a significant SNP within the *PLC*_*z*_ gene was found in Duroc pigs, related to both male and female fertility. This SNP was not significant in Landrace, however, a SNP within the *COX-2* gene was found significant for male and female fertility in Landrace.

The zona pellucida protein 3 (*ZP3*) has been reported to initiate the sperm binding and to induce the acrosome reaction in sperm cells in humans [13]. In this study, a significant SNP within the *ZP3* gene was found associated with male fertility in Duroc.

A significant SNP was found associated to male fertility in *VWF* in Duroc. *VWF* has been suggested to have an effect in the angiogenesis in the porcine oviduct in response to seminal plasma, to be involved in the early stage of endothelium activation and to be sign of vascular bed remodelling in the oviduct [14, 15]. No association with female fertility was found, despite the previously association to sperm DNA fragmentation [10].

The SNPs explained between 0.27 and 1.18% of the genetic variance for TNB. The low genetic effects for TNB is supported by other association studies for litter size in pigs [16, 17]. In highly selected breeds such as Landrace, any gene variant with a major effect on fertility is expected to be fixed. Also, the moderate heritability for litter size [e.g. 18] indicate a true quantitative nature of this traits. Only a subset of possible candidate genes was included in this study, so there might be other genes with larger effects. However, GWAS-studies in pigs have so far not identified any major QTL related to fertility [19]. The genetic effects obtained in this study are too low to be used for selection purposes directly, but might be useful in a whole genome SNP-panel for genomic selection, since these SNPs would be located closer to causal variants than SNPs mainly selected to be evenly spaced SNP across the genome.

The current study suggests a role of the genes *BMPR1*, *COX-2, PLC*_*z*_*, VWF* and *ZP3* in total number of piglets born. The SNPs explain between 0.27 and 1.18% of the genetic variance for TNB, suggesting a limited contribution to the total genetic variation of the trait. Furthermore, significant SNP-effects from fixed effects models are generally overestimated, which probably means that the explained variation is overestimated. Further studies are needed to discover the functional mutations and to find other genes contributing to the genetic variation of litter size. The SNPs obtained might, however, be of interest in SNP-panels used for genomic selection, since they are likely more associated to these traits than randomly selected SNP-markers.

## Supplementary information

**Additional file 1.** Overview over 14 genes used for association between SNP within candidate genes and fertility in Landrace and Duroc pigs.

**Additional file 2.** Primer sequences.

## Data Availability

The datasets used and/or analysed during the current study are available from the corresponding author on reasonable request.

## Abbreviations

BMP15: bone morphogenic protein 15
BMPR1B: bone morphogenic protein receptor 1B
COX-2: cyclooxygenase isoenzyme type 2
EBV: estimated breeding value
ESR1: estrogen receptor 1
ESR2: estrogen receptor 2
GDF9: growth differentiation factor
HWE: Hardy–Weinberg equilibrium
MAF: minor allele frequency
PLCz: phospholipase C zeta
SNP: single nucleotide polymorphism
TNB: total number of piglets born
VWF: Von Willenbrand factor
ZP3: zona pellucida protein 3
