# Supplementary material for: Association between single-nucleotide polymorphisms within candidate genes and fertility in Landrace and Duroc pigs
Source: Acta Vet Scand. 2019 Dec 3;61:58. doi: 10.1186/s13028-019-0493-x (PMC6888942; doi:10.1186/s13028-019-0493-x)
Supplement: Supplementary file 1 — Additional file 1. Overview over 14 genes used for association between SNP within candidate genes and fertility in Landrace and Duroc pigs. [file 13028_2019_493_MOESM1_ESM.docx]

**Additional file 1 Overview over 14 genes used for association between SNP within candidate genes and fertility in Landrace and Duroc pigs**

| \| **Gene name** \| **Abbreviation** \| \| --- \| --- \| \| Steroid 5α-reductase \| *SRD5A* \| \|  \|  \| \| Phospholipase C zeta \| *PLCz* \| \|  \|  \| \| Cyclooxygenase isoenzyme type 2 \| *COX-2* \| \|  \|  \| \| β-actin \| *ACTB* \| \|  \|  \| \| Catsper \| *CATSPER* \| \|  \|  \| \| Antrogen receptor \| *AR* \| \|  \|  \| \| Sperm protamine 1 \| *PRM1* \| \|  \|  \| \| Estrogen receptor 1 \| *ESR1* \| \|  \|  \| \| Estrogen receptor 2 \| *ESR2* \| \|  \|  \| \| Growth differentiation factor 9 \| *GDF9* \| \|  \|  \| \| Zona pellucida glycoprotein 3 \| *ZP3* \| \|  \|  \| \| Cluster-of-differentiation antigen 9 \| *CD9* \| \|  \|  \| \| Bone morphogenic protein 15 \| *BMP15* \| \|  \|  \| \| Bone morphogenic protein receptor 1B \| *BMPR1B* \| |
| --- | --- | --- | --- | --- | --- | --- | --- | --- | --- | --- | --- | --- | --- | --- | --- | --- | --- | --- | --- | --- | --- | --- | --- | --- | --- | --- | --- | --- | --- | --- | --- | --- | --- | --- | --- | --- | --- | --- | --- | --- | --- | --- | --- | --- | --- | --- | --- | --- | --- | --- | --- | --- | --- | --- | --- | --- |
