# Supplementary material for: Association between single-nucleotide polymorphisms within candidate genes and fertility in Landrace and Duroc pigs
Source: Acta Vet Scand. 2019 Dec 3;61:58. doi: 10.1186/s13028-019-0493-x (PMC6888942; doi:10.1186/s13028-019-0493-x)
Supplement: Supplementary file 2 — Additional file 2. Primer sequences. [file 13028_2019_493_MOESM2_ESM.docx]

**Additional file 2 Primer sequences**

| **Gene** | **Exon** | **F/R** | **Sequence** | **Tm** | **% GC** | **Lenght** | **ANY** | **SELF** | **3' stability** | **F/R** | **Sequence** | **Tm** | **% GC** | **Lenght** | **ANY** | **SELF** | **3' stability** |
| --- | --- | --- | --- | --- | --- | --- | --- | --- | --- | --- | --- | --- | --- | --- | --- | --- | --- |
| **ZP3** | Ex1 | F | TGGCCTCTAACATTCCCTGT | 59.5 | 50 | 20 | 4 | 1 | 7.9 | R | CCCACCTCCAACAGCTACAT | 60 | 55 | 20 | 4 | 2 | 5.6 |
|  | Ex2 | F | CCCAGTGTCTCTGGTCCATT | 60 | 55 | 20 | 7 | 1 | 8.4 | R | AGCTGTGGGCTGAACAGATT | 59.9 | 50 | 20 | 4 | 1 | 6.6 |
|  | Ex3-5 | F | GGTCAGGCTGAGACTTGAGG | 60 | 60 | 20 | 7 | 1 | 8.2 | R | TACCAAGTCGCCGAATAAGG | 60.1 | 50 | 20 | 5 | 3 | 7.5 |
|  | Ex6 | F | ACCACTGCACCACCTAGGAC | 60 | 60 | 20 | 6 | 2 | 7.6 | R | GTCAGCTTTTCCACCCAGTC | 59.7 | 55 | 20 | 4 | 1 | 6.4 |
|  | Ex7 | F | GACCCTGGTCCTTCCTTCTC | 60 | 60 | 20 | 6 | 0 | 6.7 | R | GACTGCATCCCCCAGAGTAA | 60.1 | 55 | 20 | 4 | 1 | 5.7 |
|  | Ex8 | F | AGTTCTGACCCGTCATCAGG | 60.1 | 55 | 20 | 6 | 2 | 8.2 | R | AAATGCAGGCCTGTCTTCAC | 60.3 | 50 | 20 | 8 | 2 | 6.7 |
|  |  |  |  |  |  |  |  |  |  |  |  |  |  |  |  |  |  |
| **ESR2** | Ex1 | F | TGTCCCTTTGTGCCTCTTCT | 59.8 | 50 | 20 | 2 | 0 | 6.7 | R | TGATTTGAGTAATGCCCATGAA | 60.3 | 36.4 | 22 | 4 | 2 | 6.9 |
|  | Ex2 | F | GCTTGATCGGATTTCTTGGA | 60.2 | 45 | 20 | 5 | 1 | 8.5 | R | GGTCACCTGCGAATTTGTTT | 60 | 45 | 20 | 5 | 0 | 7 |
|  | Ex3 | F | TCCCCTGTCTCTCAGCTTGT | 60 | 55 | 20 | 4 | 0 | 6.7 | R | TCCCATGTCTCCTCTCATCC | 60 | 55 | 20 | 4 | 0 | 8.1 |
|  | Ex4 | F | AGTGACTGGGGGAACTTGTG | 60 | 55 | 20 | 5 | 0 | 7 | R | CTGGTGGACCTCCATCTTGT | 60 | 55 | 20 | 7 | 0 | 6.7 |
|  | Ex5 | F | CTTCCTTGGCAGCATTTAGC | 60 | 50 | 20 | 4 | 2 | 7.5 | R | ATGCTCTCCTTCTTCGGTGA | 60 | 50 | 20 | 3 | 2 | 7.9 |
|  | Ex6 | F | TTCTGGCTGTGGTCTCATTG | 59.8 | 50 | 20 | 3 | 3 | 7.2 | R | GAAAGGCATTTTCCCCTTTT | 59.4 | 40 | 20 | 6 | 1 | 7.3 |
|  | Ex7 | F | CTGAACAACCCAGGTCCACT | 60 | 55 | 20 | 4 | 1 | 7.9 | R | AAACTTGCACAATCCGTTCC | 60 | 45 | 20 | 4 | 0 | 7.9 |
|  | Ex8 | F | GTGAACAGCAAGACCCCATT | 60 | 50 | 20 | 2 | 1 | 8.4 | R | AGTCCACCCACTGCTTATGG | 60 | 55 | 20 | 3 | 0 | 7.4 |
|  |  |  |  |  |  |  |  |  |  |  |  |  |  |  |  |  |  |
| **GDF9** | Ex1 | F | GAGGCTGAACAGGCGATAAA | 60.4 | 50 | 20 | 4 | 0 | 6.2 | R | TCAAGTACCGCATTTGCTTG | 59.9 | 45 | 20 | 5 | 2 | 8.5 |
|  | Ex2 | F | AGAAAGGGTTCACGAAAGCA | 59.9 | 40 | 20 | 4 | 0 | 8.5 | R | CCATTGAAGGAGCAGGGTTA | 60.1 | 50 | 20 | 3 | 2 | 7.2 |
|  |  |  |  |  |  |  |  |  |  |  |  |  |  |  |  |  |  |
| **PLCz** | Ex1 | F | GGTGTTCAGACCGAAAGGAA | 60.1 | 50 | 20 | 5 | 0 | 8.2 | R | CAGTGGGTTGGAGGAAGCTA | 60.2 | 55 | 20 | 4 | 2 | 7.2 |
|  | Ex2 | F | TGCAGGGACCACATCTTACA | 60.1 | 50 | 20 | 4 | 1 | 6 | R | TCAGGGCCTTTCAAAAAGAA | 59.8 | 40 | 20 | 5 | 1 | 7 |
|  | Ex3 | F | CATGAGATAGACTGCCCTCTGA | 59.5 | 50 | 22 | 4 | 3 | 6.7 | R | TCCTTCCTAGGGTGGAGTGG | 61.4 | 60 | 20 | 8 | 0 | 7.9 |
|  | Ex4 | F | TTCACAATATGAGCCCACCA | 59.9 | 45 | 20 | 5 | 0 | 8.2 | R | CCAGAAGAAATGTGACCCTGT | 59.1 | 47.6 | 21 | 3 | 1 | 7.9 |
|  | Ex5 | F | GTCTTCTGGGGTGCTGTCAT | 60.1 | 55 | 20 | 3 | 2 | 6.3 | R | GAGGGTGGCAGACAGGATTA | 60.1 | 55 | 20 | 3 | 2 | 5.9 |
|  | Ex6 | F | GAAGAAGAGGGAGGGAGGAA | 60.1 | 55 | 20 | 0 | 0 | 8.2 | R | GGCCAAGCATTACAGGATTT | 59 | 45 | 20 | 4 | 0 | 6.9 |
|  | Ex7 | F | TGCTCTACCTCCTTTGTGACAG | 59 | 45 | 20 | 5 | 3 | 6.4 | R | TTCAGCTGAGCTTCTCAGACA | 59 | 47.6 | 21 | 8 | 3 | 6.4 |
|  | Ex8 | F | GCTTTGGGCAAATTGAAAAT | 59.1 | 35 | 20 | 6 | 3 | 7.2 | R | TTAGACACCAGCCTGGGAAC | 60.1 | 55 | 20 | 7 | 3 | 7.9 |
|  | Ex10 | F | TGTGAACCATAAAGGCATGAA | 59 | 38.1 | 21 | 4 | 0 | 6.9 | R | CAAAACACCTAGGGCCAAAA | 60 | 45 | 20 | 6 | 0 | 7.6 |
|  | Ex11 | F | GACATGGCAGAAGGAAATGTT | 59 | 42.9 | 21 | 4 | 2 | 6.6 | R | TGCTGAAATACCACCCACCT | 60.4 | 50 | 20 | 2 | 0 | 7.9 |
|  | Ex12 | F | TTTTGGTGTGTGGGAATTGA | 59.8 | 40 | 20 | 4 | 1 | 6.9 | R | TTGCTGCATATAACACATTGGTC | 59.9 | 39.1 | 23 | 5 | 1 | 7.9 |
|  | Ex13 | F | TCCCCTAGGGAGAGTGTTTG | 59.1 | 55 | 20 | 8 | 0 | 7 | R | CCCCAACTCCACCTTCTAAA | 59 | 50 | 20 | 2 | 2 | 6.3 |
|  | Ex14 | F | TGGAGATGCTGCATTTTGAG | 59.9 | 45 | 20 | 7 | 1 | 7 | R | CTGGCCTGAGATTTCCCATA | 60 | 50 | 20 | 4 | 2 | 7.4 |
|  |  |  |  |  |  |  |  |  |  |  |  |  |  |  |  |  |  |
| **SRD5A2** | Ex1 | F | CCAGTCCTGGGTTTGTAGGA | 60 | 55 | 20 | 6 | 0 | 7.2 | R | AACTGGGTCGTGGTGAGAAG | 60.2 | 55 | 20 | 2 | 0 | 6.7 |
|  | Ex2 | F | GCACAAATACGCTTTGCTGA | 60 | 45 | 20 | 4 | 2 | 8.2 | R | CGAGGAAGAGTGGGAATCTG | 59.8 | 55 | 20 | 3 | 1 | 6.6 |
|  | Ex3 | F | TTGCTGGTTCTTCTGCACAC | 60 | 50 | 20 | 4 | 2 | 6.4 | R | ACTTGGGTTTGGTTGTGCTC | 60 | 50 | 20 | 2 | 2 | 8.2 |
|  | Ex4-1 | F | TGACTGCAGGCTGATTTGTC | 60 | 50 | 20 | 6 | 1 | 6.7 | R | CATCCCTCTGGTTGCAGTTT | 60.1 | 50 | 20 | 4 | 0 | 6.7 |
|  | Ex4-2 | F | GCGGTCTTAGTGCCTCTGTC | 60 | 60 | 20 | 3 | 1 | 6.4 | R | GACATCTGAGCTGTGGACGA | 60 | 55 | 20 | 7 | 1 | 8.1 |
|  |  |  |  |  |  |  |  |  |  |  |  |  |  |  |  |  |  |
| **AR** | Ex1 | F | GTTGCATTTGCTTTCCACCT | 60.1 | 45 | 20 | 4 | 0 | 7.9 | R | AGGGAGTCACCTCTCTGCAA | 60 | 55 | 20 | 5 | 2 | 8.8 |
|  | Ex2-1 | F | ACCCCTTCAAGATTTGAGCA | 59.7 | 45 | 20 | 6 | 2 | 8.2 | R | CGACTCGGATAGGCTGCTAC | 60 | 60 | 20 | 5 | 1 | 6.9 |
|  | Ex3 | F | TGGGAGAATTCCAAGGAAAA | 59.5 | 40 | 20 | 6 | 2 | 7.3 | R | GGGGACCAGCAAGTATGAAA | 59.9 | 50 | 20 | 3 | 1 | 7.3 |
|  | Ex4 | F | CCTGGGAACCTTCATATGCT | 59 | 60 | 20 | 6 | 2 | 8.1 | R | GGAAAGTAAAGGGGGAAGGA | 59.4 | 50 | 20 | 2 | 0 | 8.2 |
|  | Ex5 | F | GCACTTGCCCTTAAAGGAGTT | 59.8 | 47.6 | 21 | 7 | 2 | 6.4 | R | TGGTCCATAGGTGCATTCAA | 59.9 | 45 | 20 | 5 | 3 | 7.3 |
|  | Ex6 | F | CACTGCCTCTGCCCTATCTC | 60 | 60 | 20 | 3 | 0 | 6.3 | R | GTCACCCAGGTCTGGACAGT | 60 | 60 | 20 | 6 | 3 | 6.1 |
|  | Ex7 | F | CCTGTCTCAGGAAGCAGGAG | 60.1 | 60 | 20 | 6 | 2 | 7.9 | R | AGCTCCCCTTTGATTCCCTA | 60 | 50 | 20 | 4 | 2 | 8.7 |
|  | Ex8 | F | CTAGCCTTTTCCCCAAGACC | 60.1 | 55 | 20 | 4 | 0 | 7.6 | R | ACTCAAAGCCAGAGGGGAAT | 60.1 | 50 | 20 | 3 | 2 | 8.1 |
|  |  |  |  |  |  |  |  |  |  |  |  |  |  |  |  |  |  |
| **CATSPER1** | Ex2 | F | CTCAGGCAGACCTCAAGTCC | 60 | 60 | 20 | 5 | 3 | 7.6 | R | TCACTCTAAGGCTGGGCAAT | 59.8 | 50 | 20 | 3 | 2 | 8.4 |
|  | Ex8-9 | F | CACGAATCCAAAGGAGGGTA | 59.9 | 50 | 20 | 3 | 2 | 8.4 | R | TTTTAGGGCCATACCCACAG | 59.8 | 50 | 20 | 6 | 1 | 6.7 |
|  | Ex10 | F | ACTAGTCGGGTTCGTCATCG | 60.1 | 55 | 20 | 6 | 2 | 8.6 | R | GGCACAAGGTGACTCTGGTT | 60.2 | 55 | 20 | 5 | 3 | 8.2 |
|  | Ex11-12 | F | ATAATCGTCGGAGGCTGTGT | 59.6 | 50 | 20 | 3 | 1 | 6.4 | R | CAGCAGATCTGGGGACTTGT | 60.3 | 55 | 20 | 8 | 2 | 6.7 |
|  |  |  |  |  |  |  |  |  |  |  |  |  |  |  |  |  |  |
| **BMP15** | Ex1 | F | ATTTTGGCTTGTGCTGGAAC | 60.1 | 45 | 20 | 2 | 2 | 7.9 | R | CCCAAGGCAATCTCTCGTTA | 60.2 | 50 | 20 | 2 | 2 | 7.7 |
|  | Ex2 | F | GATCCTCAACCCACCGAGTA | 59.9 | 55 | 20 | 4 | 2 | 5.4 | R | CCCATAGATTTGGCAGAGGA | 60 | 50 | 20 | 4 | 0 | 7.9 |
|  |  |  |  |  |  |  |  |  |  |  |  |  |  |  |  |  |  |
| **ESR1** | Ex1 | F | AGGGATTCTTGGAACCTTGG | 60.3 | 50 | 20 | 3 | 2 | 8.5 | R | CTTTCTTCCTTGGCCTTCCT | 59.8 | 50 | 20 | 4 | 0 | 8.2 |
|  | Ex2 | F | GAGCGGTACTCTGGCATCAG | 61 | 60 | 20 | 4 | 2 | 6.6 | R | CGCTTTCTCACGCTCTCTCT | 60 | 55 | 20 | 2 | 0 | 6.4 |
|  | Ex3 | F | TGTGATTGTGCCTTCAGAGC | 60 | 50 | 20 | 3 | 2 | 7.9 | R | ATGAAGAGAATGGGCTGTGG | 60.1 | 50 | 20 | 2 | 0 | 8.2 |
|  |  |  |  |  |  |  |  |  |  |  |  |  |  |  |  |  |  |
| **BMPR1B** | Ex1-1 | F | CAGAAACCCTCCATCCTTCA | 60 | 50 | 20 | 3 | 1 | 7 | R | CAGCTTTCAGGGGATATGGA | 60 | 50 | 20 | 4 | 0 | 8.1 |
|  | Ex1-2 | F | CCCTCCCTCCCTTAACTCAG | 60.1 | 60 | 20 | 4 | 3 | 6.7 | R | TTGACCATTGGCATGAGAAA | 60 | 40 | 20 | 5 | 2 | 7 |
|  | Ex2 | F | TCACAACGACCTGGTGTCAT | 60 | 50 | 20 | 5 | 3 | 6.3 | R | TGGAGCCCAGTTGGTTAGAC | 60.1 | 55 | 20 | 4 | 2 | 5.4 |
|  | Ex3 | F | TTTCTGGTCAGCGAGGACTT | 60 | 50 | 20 | 4 | 1 | 6.4 | R | AATCCCCTTGACTGGTCCTT | 59.8 | 50 | 20 | 4 | 0 | 8.2 |
|  | Ex4 | F | TTCCTTCTTTTTCCCTGCAA | 59.8 | 40 | 20 | 4 | 2 | 8.8 | R | AACAAGGAGCTCCGAACCTT | 60.2 | 50 | 20 | 8 | 3 | 7.9 |
|  | Ex5 | F | CTGTGAGGGCTCCCATAAGA | 60.2 | 55 | 20 | 6 | 2 | 6 | R | TTCAACAAATCTGTCGTGCTG | 59.9 | 42.9 | 21 | 6 | 1 | 8.5 |
|  | Ex6 | F | CCTCTGCCTCAGGAGTAGGA | 59.5 | 60 | 20 | 7 | 3 | 7.2 | R | TGGGTTAAGCCACACCTTCT | 59.6 | 50 | 20 | 8 | 0 | 6.7 |
|  | Ex7 | F | ATCTGGCCAATGAAAGGTTG | 59.9 | 45 | 20 | 6 | 0 | 8.2 | R | GGTACCTGCGAAAGACCAAA | 60.1 | 50 | 20 | 6 | 0 | 8.8 |
|  | Ex8 | F | GTGCGGCTCCATGAGTTAAT | 60.1 | 50 | 20 | 4 | 2 | 6.2 | R | GCTGGGCTATCCCACAGATA | 60.1 | 55 | 20 | 8 | 3 | 5.6 |
|  | Ex9 | F | TCCTGATCAGCTTGAACGTG | 60 | 50 | 20 | 8 | 3 | 8.1 | R | AACACAGACCTGTGCCATGA | 60.2 | 50 | 20 | 8 | 2 | 6.9 |
|  | Ex10 | F | CTTCACGAGCACCTCCTCTC | 60.1 | 60 | 20 | 4 | 0 | 6.4 | R | TTATGTTGCCTCCAGTCGTG | 59.7 | 50 | 20 | 2 | 1 | 8.4 |
|  |  |  |  |  |  |  |  |  |  |  |  |  |  |  |  |  |  |
| **COX-2** | Ex1-1 | F | CAGGGCCAGTTTGGAATAGA | 60.1 | 50 | 20 | 4 | 0 | 5.6 | R | GAACTGAGATCCCCCATCAA | 59.9 | 50 | 20 | 5 | 0 | 6.9 |
|  | Ex1-2 | F | CTGCAGCCTTAAACCCAGTT | 59.4 | 50 | 20 | 6 | 3 | 6.7 | R | AGGCTGCCCTTTTACCTCAT | 60.1 | 50 | 20 | 5 | 2 | 6.6 |
|  | Ex1-3 | F | CAGCATTTTGCCGTCTTGTA | 59.9 | 45 | 20 | 3 | 2 | 6 | R | TGAGCGGAGGGGTAAAACTA | 57.9 | 50 | 20 | 2 | 2 | 5.7 |
|  | Ex2 | F | GCTTTCCTCTCCTGTTTCCA | 59.4 | 50 | 20 | 2 | 0 | 8.5 | R | TCTTTGTACCCAAGGGATGC | 59.9 | 50 | 20 | 4 | 2 | 8.1 |
|  | Ex3 | F | CTGACTAAGCCCTTTGTTTCG | 59 | 47.6 | 21 | 6 | 2 | 9 | R | CCCTGGGCTTGATTAGGAAT | 60.3 | 50 | 20 | 5 | 3 | 8.1 |
|  | Ex4 | F | TTCCCTGGAGAAGAGCGTTA | 59.9 | 50 | 20 | 5 | 2 | 7.7 | R | CAGCATCATTAACGATTGGAA | 58.6 | 38.1 | 21 | 8 | 2 | 8.5 |
|  | Ex5 | F | CAATTTAAACCTCCCCCGATA | 60 | 42.9 | 21 | 6 | 2 | 7.6 | R | TTCATTATTTTGTGGCTGTGAAA | 59.5 | 30.4 | 23 | 4 | 1 | 7.3 |
|  | Ex6 | F | TGGCCTTTAATGTTCACACTTC | 59.1 | 40.9 | 22 | 4 | 2 | 6.4 | R | TGACAGCCACTCTCTGCTTC | 59.3 | 55 | 20 | 3 | 0 | 8.2 |
|  | Ex8 | F | TTGAGGCAGCTAAAAACCCTA | 59 | 42.9 | 21 | 4 | 2 | 8.7 | R | GCACCCGAACAGGATTCTAC | 59.6 | 55 | 20 | 4 | 0 | 5.4 |
|  | Ex9 | F | ATTCCAAACAAGGGCACTCA | 60.5 | 45 | 20 | 4 | 1 | 6.4 | R | CACCACGGGAACTCTGGTAT | 59.8 | 55 | 20 | 4 | 2 | 6.8 |
|  |  |  |  |  |  |  |  |  |  |  |  |  |  |  |  |  |  |
| **ACTB** | Ex3 | F | TGTTTCTGCCTGCTTCCTTT | 60 | 45 | 20 | 2 | 0 | 8.5 | R | GAGGTCCTTCCTGATGTCCA | 60 | 55 | 20 | 5 | 2 | 7.9 |
|  | Ex4 | F | CTCTTCCAGCCCTCCTTCTT | 60 | 55 | 20 | 2 | 0 | 7 | R | GAGCCGCGTGTGTGTAACTA | 59.9 | 55 | 20 | 4 | 2 | 5.7 |
|  | Ex5 | F | ACGTGGACATCAGGAAGGAC | 60 | 55 | 20 | 4 | 1 | 7.6 | R | TGGTTTTCCCAAGTCAGACC | 59.9 | 50 | 20 | 5 | 3 | 7.6 |
|  |  |  |  |  |  |  |  |  |  |  |  |  |  |  |  |  |  |
| **CD9** | Ex2 | F | CACGCAGTACCCCTTCTCTC | 59.9 | 60 | 20 | 4 | 0 | 6.4 | R | GGCTTGGTCAACTTGGAAAA | 60.1 | 45 | 20 | 5 | 3 | 7.3 |
|  | Ex3 | F | CATCTCCTCGTCCTGTCCTC | 59.8 | 60 | 20 | 2 | 0 | 7.9 | R | GAGACAGAGCCCTGCTCAAG | 60.3 | 60 | 20 | 8 | 3 | 7 |
|  | Ex4 | F | ACACCTCACCTCAGCCCTTT | 61.1 | 55 | 20 | 3 | 0 | 8.5 | R | ATTGAAGGCTGACCCAAGTG | 60.1 | 50 | 20 | 3 | 2 | 6.7 |
|  | Ex5 | F | GTGGCTGAGCATGAATGAGA | 60 | 50 | 20 | 5 | 0 | 6.7 | R | AAGCAAGGGGAGGAGAGAAG | 60 | 55 | 20 | 2 | 0 | 6.7 |
|  | Ex6 | F | TTGTCTCTCTCACCCCATCC | 60 | 55 | 20 | 2 | 0 | 8.1 | R | ATGGTGGGTCCTTCCTGAG | 59.9 | 57.9 | 19 | 3 | 3 | 6.7 |
|  | Ex7 | F | CGTGTGTGACCTGGATCTTG | 60.2 | 55 | 20 | 4 | 2 | 7 | R | CCCCAAAACAAAAGCAAAAA | 60 | 35 | 20 | 2 | 0 | 7.6 |
|  |  |  |  |  |  |  |  |  |  |  |  |  |  |  |  |  |  |
| **PRM1** | Ex1 | F | CAAAGTTCCACCTGCTCACA | 59.9 | 50 | 20 | 3 | 1 | 6.7 | R | AGTGCGGTGGTCTTGCTACT | 59.9 | 55 | 20 | 3 | 1 | 5.4 |
|  | Ex2 | F | TCACCATGGCCAGATACAGA | 60.1 | 50 | 20 | 6 | 0 | 6.4 | R | CAAGGCAAATGAGACCAGTG | 59.3 | 50 | 20 | 3 | 3 | 6.7 |
